# Supplementary material for: Paradoxical mTORC1-Dependent microRNA-mediated Translation Repression in the Nucleus Accumbens of Male Mice Consuming Alcohol Attenuates Glycolysis
Source: Nat Commun. 2025 Jul 14;16:6116. doi: 10.1038/s41467-025-60337-9 (PMC12259992; doi:10.1038/s41467-025-60337-9)
Supplement: Supplementary file 1 — Supplementary Information [file 41467_2025_60337_MOESM1_ESM.pdf]

Supplementary Figure 1: Alcohol does not alter GAPDH mRNA and protein levels in the NAc

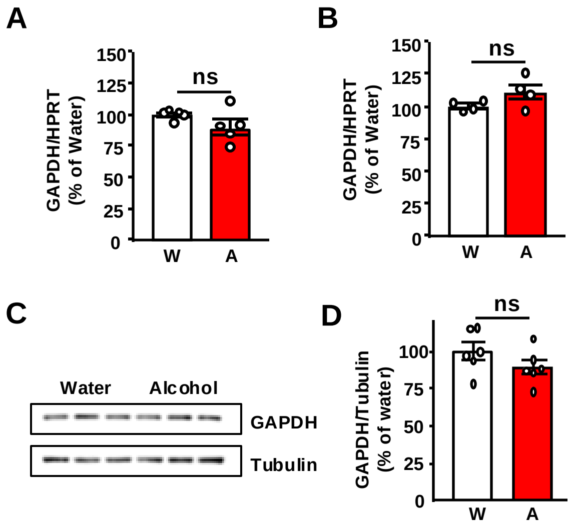

Supplementary Figure 1 Alcohol does not alter GAPDH mRNA levels in the NAc

(**A-B**) Polysomal mRNA levels of GAPDH in D1+ neurons (**A**), and D2+ neurons (**B**) after alcohol withdrawal were determined by RT-qPCR. Data are presented as the average ratio of a transcript to HPRT±SEM and expressed as % of water control. ns: non-significant. n = 4 per group. Significance was determined using two-tailed Mann-Whitney t-test. (**A**) U=5, p=0.1508; (**B**) U=5, p=0.2.

(C) GAPDH protein levels in the NAc. (**D**) Data are presented as the average ratio of GAPDH to Tubulin±SEM and are expressed as % of water control. ns: non-significant. n = 6 per group. Significance was determined using two-tailed Mann-Whitney t-test. U=9, p=0.1797.

Source data are provided as a Source Data file.

Supplementary Figure 2: Alcohol does not alter the transcription of Trax and GW182 in the NAc and the protein levels of GW182 and Trax in the DLS

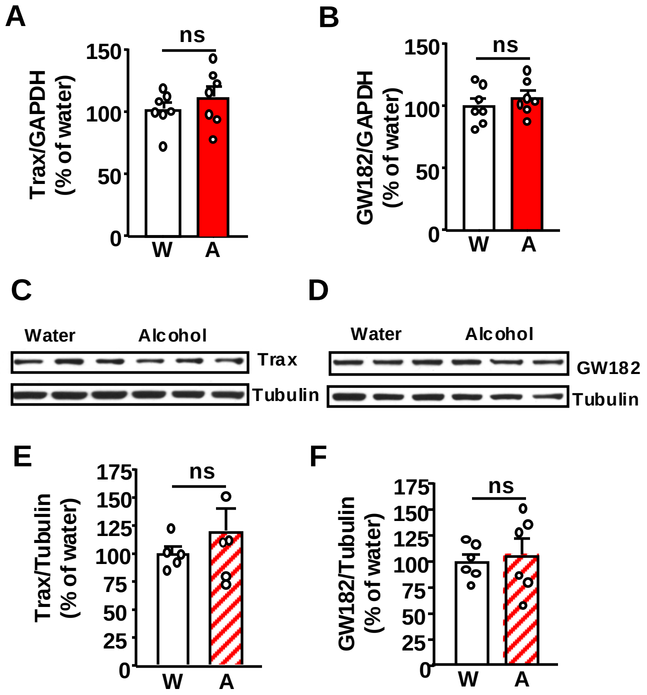

Supplementary Figure 2 Alcohol does not alter the transcription of Trax and GW182 in the NAc and the protein levels in the DLS

After 7 weeks of IA20%2BC (**Supplementary Table 1**), the NAc of mice was dissected at the end of the 24 hours alcohol withdrawal session. Control animals had access to water only. **(A-B)** GW182 and Trax mRNA level was determined by RT-qPCR in the total mRNA sample. Data are presented as the average ratio of GW182 or Trax to GAPDH±SEM and expressed as % of water control. ns: non-significant. n = 7 per group. Significance was determined using two-tailed Mann-Whitney t-test. (**A**) U=18, p=0.4557; (**B**) U=17, p=0.3829.

(C-D) Trax and GW182 protein levels in the DLS. (**E-F**) Data are presented as the average ratio of GW182 or Trax to Tubulin±SEM and are expressed as % of water control. ns: non-significant. (**E**) water: n = 5; alcohol: n = 6 (**F**) n = 6 per group. Significance was determined using two-tailed Mann-Whitney t-test. (**E**) U=16, p=0.8182; (**F**) U=12, p=0.6623.

Source data are provided as a Source Data file.

Supplementary Figure 3: Categories of transcripts that are reduced by alcohol in an mTORC1 dependent

manner

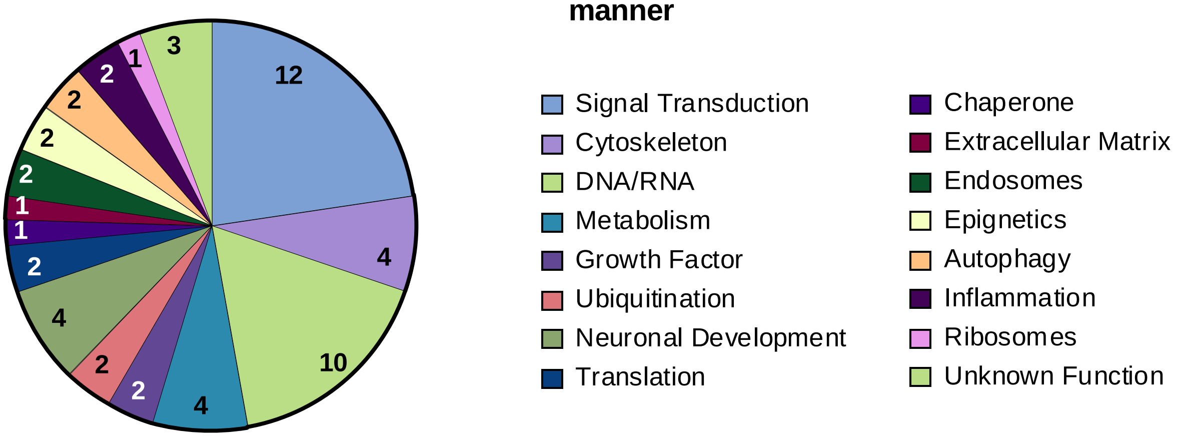

Signal Transduction
Klhl3 (Kelch-like 3)
Pdlim1(PDZ and Lim domain 1)
Ccdc93 (coiled-coil domain containing 93)
Flt1 (FMS-like tyrosine kinase 1)
PPM1E Protein phosphatase 1E
Pde4dip (phosphodiesterase 4D interacting protein)
Robo3 (Roundabout guidance receptor 3)
Tsc1 (tuberous sclerosis 1)
Igtp (Interferon gamma induced GTPase)
Dennd3 (DENN Domain Containing 3)
RIOK2 (RIO Kinase 2)
bHlha9 (basic helix loop helix family member a9)

DNA/RNA
Inip (INTS3 and NABP interacting protein)
TSEN34 (TRNA Splicing Endonuclease Subunit 34)
Rbfox1 (RNA binding protein fox1 homolog)
Tyw5 (tRNA-wybutosine synthesizing protein 5)
WBSCR27 (Williams–Beuren syndrome-related methyltransferase)
Atxn7l3 (Ataxin 7-like 3)
bHLHA9 (basic helix loop helix family member a9)
Neil 1 (Nei endonuclease VIII-like 1)
Zranb3 (zinc finger RNA-binding domain containing 3)
bHlha9 (basic helix loop helix family member a9)

Actin/Cytoskeleton
XIRP2 Xin actin-binding repeat-containing protein 2
Pdlim1 (PDZ and Lim domain 1)
Epb41l4a (erythrocyte membrane protein band 4.1 like 4a)
Abi3 (ABI gene family member 3)

Metabolism
Protein phosphatase 1E (PP2C domain containing) (PPM1E)
Aldolase A
Ptgs2 (Prostaglandin endoperoxide synthase 2)
HS3ST4 (Heparan sulfate-glucosamine 3-sulfotransferase 4)

Growth Factor
Lcn2 (lipocalin) neutrophil gelatinase-associated lipocalin (NGAL)
Flt1 (FMS-like tyrosine kinase 1)

Ubiquitination
Klhl3 (Kelch-like 3)
Atxn7l3 (Ataxin 7-like 3)

Neuronal Development
Robo3 (Roundabout guidance receptor 3)
Neil 1 (Nei endonuclease VIII-like 1)
Vwa1 (von Willebrand factor A domain containing 1)
Ptgs2 (Prostaglandin endoperoxide synthase 2)

Translation
Tsc1 (tuberous sclerosis 1)
RIOK2 (RIO Kinase 2)

Chaperone
Clgn (calmegin)

Extracellular Matrix
Vwa1 (von Willebrand factor A domain containing 1)

Endosomes
Clgn (calmegin)
Ccdc93 (coiled-coil domain containing 93)

Epigenetics
Atxn7l3 (Ataxin 7-like 3)
Tyw5 (tRNA-wybutosine synthesizing protein 5)

Autophagy
Dennd3 (DENN Domain Containing 3)
Tsc1 (tuberous sclerosis 1)

Inflammation
Lcn2 (lipocalin) neutrophil gelatinase-associated lipocalin (NGAL)
Ptgs2 (Prostaglandin endoperoxide synthase 2)

Ribosomes
RIOK2 (RIO Kinase 2)

Unknown Function
Fam163b
Mta3 (metastasis associated 3)
Predicted gene 5128(Gm5128)

Supplementary Figure 3 Functional characterization of RNAseq transcripts whose translation are decreased by alcohol in an mTORC1-dependent manner

Functional categories were determined by an up-to-date literature search of each of the transcripts highlighting the transcripts' known function. Some proteins have several functions and are, therefore, found in multiple functional categories.

Supplementary Figure 4 Alcohol does not alter the transcription of Aldolase A, PPM1E, Rbfox2 in the NAc and their protein level in the DLS

After 7 weeks of IA20%2BC (**Supplementary Table 1**), the NAc of mice was dissected at the end of the 24 hours alcohol withdrawal session. Control animals had access to water only. **(A-C)** Total Aldolase A (**A**), PPM1E (**B**), Rbfox2 (**C**) mRNA levels were determined by RT-qPCR in the total mRNA sample. Data are presented as the average ratio of Aldolase A, Rbfox2 or PPM1E to GAPDH±SEM and expressed as % of water control. ns: non-significance. n = 7 per group. Significance was determined using two-tailed Mann-Whitney t-test. (**A**) U=19, p=0.535; (**B**) U=22, p=0.8048; (**C**) U=22, p=0.8048.

(D-F) Aldolase A, PPM1E, Rbfox2 protein levels after withdrawal were determined by western blot analysis. (**G-I**) Data are presented as the average ratio of Aldolase A, PPM1E, Rbfox2 to Tubulin±SEM and are expressed as the % of water control. ns: non-significant. n = 6 per group. Significance was determined using two-tailed Mann-Whitney t-test. (**G**) U=13, p=0.4848; (**H**) U=8, p=0.132; (**I**) U=14, p=0.5887.

Source data are provided as a Source Data file.

Supplementary Figure 4: Alcohol does not alter the transcription of Aldolase A, PPM1E and Rbfox2 in the NAc and protein level in the DLS

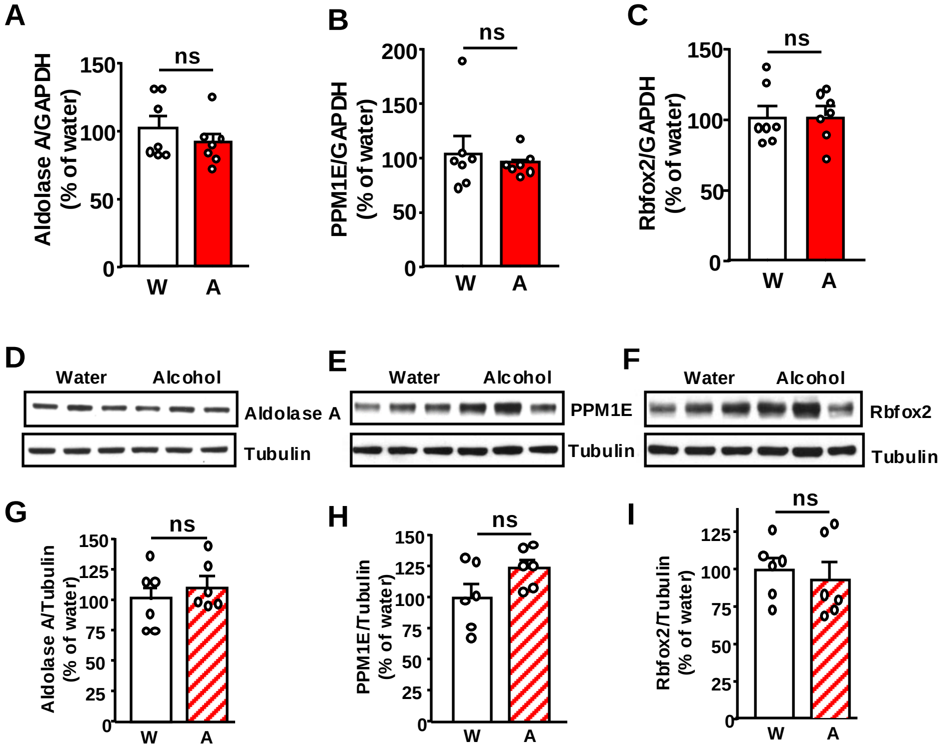

Supplementary Figure 5 Alcohol does not affect the translation of Trax, Gw182 and CNOT4, PPM1E and Rbfox2 but increases the translation of Aldolase A in D2+ neurons

(**A-F**) Polysomal mRNA levels in D2+ neurons of Trax (**A**), GW182 (**B**), CNOT4 (**C**), Aldolase A (**D**), PPM1E (**E**), Rbfox2 (**F**) after alcohol withdrawal were determined by RT-qPCR. Data are presented as the average ratio of a transcript to GAPDH±SEM and expressed as % of water control. *p<0.05, ns: non-significant. n = 4 per group. Significance was determined using two-tailed Mann-Whitney t-test. (**A**) U=6, p=0.6857; (**B**) U=6, p=0.0571; (**C**) U=6, p=0.6857; (**D**) U=6, p=0.0286; (**E**) U=6, p=0.0571; (**F**) U=6, p=0.6857.

Source data are provided as a Source Data file.

Supplementary Figure 5: Alcohol does not affect the translation of Trax, GW182 and CNOT4, PPM1E and Rbfox2 but increases the translation of Aldolase A in D2+ neurons

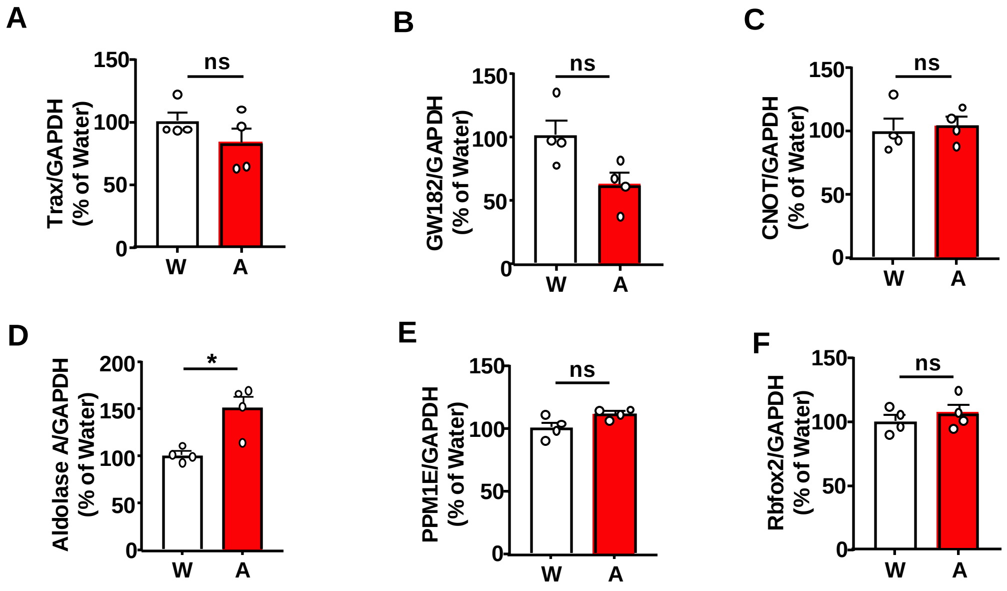

Supplementary Figure 6 U6 expression in the NAc is not altered by alcohol

After 7 weeks of IA20%2BC (**Supplementary Table 1**), the NAc of mice was dissected at the end of the 24 hours alcohol withdrawal session. Control animals had access to water only. U6 levels were measured by RT-qPCR. Data are presented as the average ratio of U6 to 5S±SEM and expressed as % of water control. ns: non-significant. n = 12 per group. Significance was determined using two-tailed Mann-Whitney t-test. U=65, p=0.7125.

Source data are provided as a Source Data file.

Supplementary Figure 6: U6 expression in the NAc is not altered by alcohol

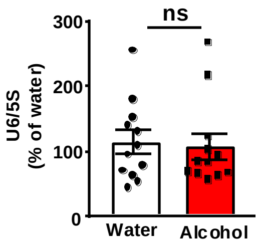

Supplementary Figure 7 The levels of miR-15b-5p, miR-25-3p, miR-92a-3p, and miR-34a-5p are not altered in the DLS After 7 weeks of IA20%2BC (**Supplementary Table 1**), the NAc of mice was dissected at the end of the 24 hours alcohol withdrawal session. Control animals had access to water only. miRs levels were measured by RT-qPCR. ns: non-significant. Data are presented as individual data points and mean ± SEM. n = 7 per group. Significance was determined using Two-way ANOVA followed by Sidak's multiple comparisons test. Alcohol x miR: F(3, 48)=0.1223, p=0.9465, effect of Alcohol: F(1, 48)=4.160, p=0.0469, Effect of miR: F(3, 48)=0.2028, p=0.8939; miR15b-5p water vs. alcohol, p=0.8127; miR 25-3p water vs. alcohol, p=0.9047; miR 34a-5p water vs. alcohol, p= 0.8868; miR 92a-3p water vs. alcohol, p=0.4289.

Source data are provided as a Source Data file.

Supplementary Figure 7: The levels of miR-15b-5p, miR-25-3p, miR-92a-3p, and miR-34a-5p are not altered by alcohol in the DLS

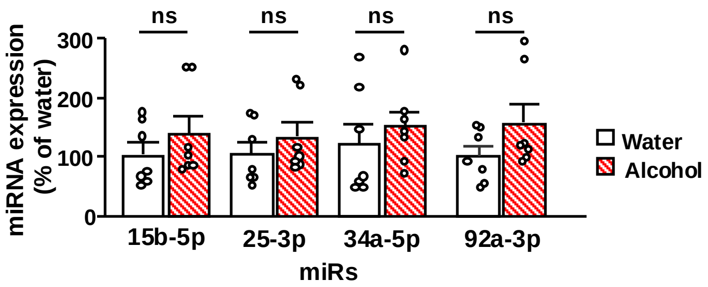

Supplementary Figure 8 Alcohol does not alter the protein levels of Aldolase C in the NAc

After 7 weeks of IA20%2BC (**Supplementary Table 1**), the NAc of mice was dissected at the end of the 24 hours alcohol withdrawal session (A, red). Control animals had access to water only (W, white). **(A)** Aldolase C level was determined by western blot analysis. (**B**) Data are presented as the average ratio of Aldolase C to Tubulin±SEM and are expressed as % of water control. ns: non-significant. n = 3 per group. Significance was determined using two-tailed Mann-Whitney t-test. U=1, p=0.2.

Source data are provided as a Source Data file.

Supplementary Figure 8: Alcohol does not alter the protein levels of Aldolase C in the NAc

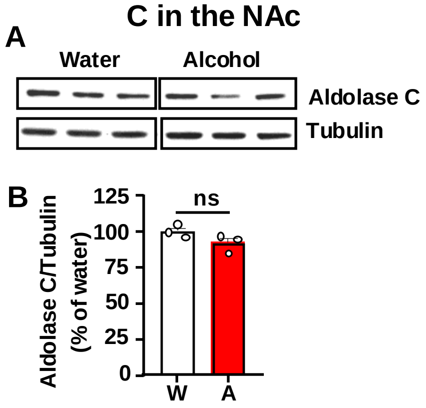

Supplementary Figure 9 Alcohol does not alter the translation of the main glucose and lactate transporters in the NAc

After 7 weeks of IA20%2BC, the NAc of mice was dissected at the end of the 24 hours alcohol withdrawal session (A, red). Control animals had access to water only (W, white). RNAseq data [19] revealed that the translation of the astrocytic glucose transporter Glu1 (**A**), the neuronal glucose transporter Glu3 (**B**), the neuronal lactate transporter MTC2 (**C)** and the astrocytic lactate transporter MTC4 **(D**) is unaltered by alcohol. W: water, A: alcohol. FPKM: Fragments per kilobase of transcript per million mapped reads. Data are presented as individual data points and mean ± SEM. ns: non-significant. n = 3 per group. Significance was determined using two-tailed Mann-Whitney t-test. (**A**) U=1, p=0.2; (**B**) U=2, p=0.4; (**C**) U=2, p=0.4; (**D**) U=2, p=0.4.

Source data are provided as a Source Data file.

Supplementary Figure 9: Alcohol does not affect the translation of the main glucose and lactate transporter in the NAc

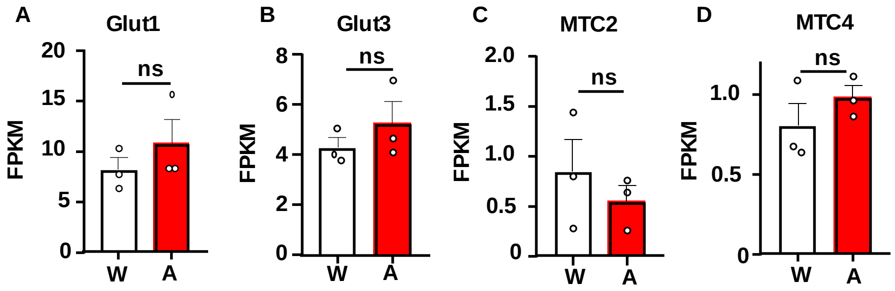

Supplementary Figure 10 Blood glucose tolerance is unaltered by chronic alcohol drinking (**A-B**) Glucose tolerance test was performed after 4 weeks (**A**) and 7 weeks (**B**) of IA20%2BC (**Supplementary Table 1**). Control animals had access to water only. Mice were subjected to 6 hours fasting before glucose (1g/kg) injection. Blood was collected before glucose injection 15-120 minutes post glucose administration, and glucose levels were determined. Data are presented as mean ± SEM. water: n = 6; alcohol: n = 12. Significance was determined using Two-way ANOVA. (**A**) Alcohol x Time: $F(4, 64)=2.232$, $p=0.0753$, effect of Alcohol: $F(1, 16)=2.397$, $p=0.1411$, effect of Time: $F(4, 64)=158.3$, $p<0.0001$. (**B**) Alcohol x Time: $F(4, 64)=1.911$, $p=0.1193$, effect of Alcohol: $F(1, 16)=3.159$, $p=0.0945$, effect of Time: $F(4, 64)=148.8$, $p<0.0001$. Source data are provided as a Source Data file.

Supplementary Figure 10: Blood glucose is unaltered by chronic alcohol drinking

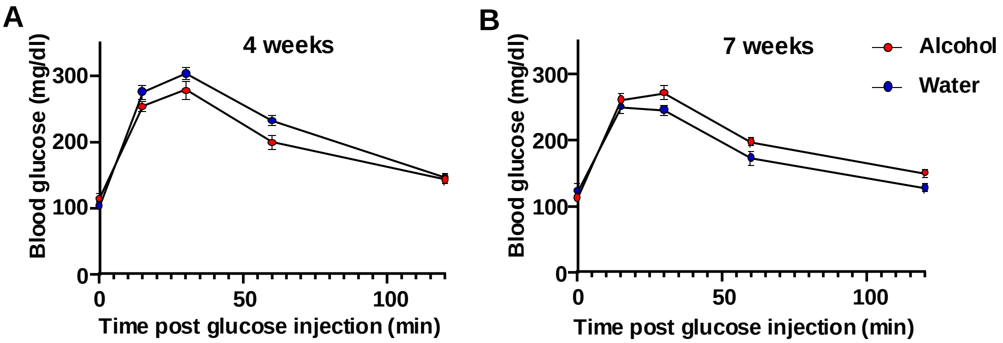

Supplementary Figure 11 Characterization of AAV2-Flex-miR-34a-GFP in cells and in the NAc

(A) N2A cells were transfected with plasmid pAAV2-Flex-miR-34a-GFP alone or combined with the plasmid pAAV-Cre. Representative images at 20x magnification of labeled GFP in green, and brightfield. Scale bar 400µm.

(B-C) AAV-Cre-dependent overexpression of miR34a in D1 NAc neurons. AAV2-Flex-miR-34a-GFP or AAV2-Flex-control was infused into the NAc of D1-Cre mice and Aldolase A protein level was evaluated by western blot analysis.

Source data are provided as a Source Data file.

Supplementary Figure 11: Characterization of AAV2-Flex-miR-34a-GFP in cells and in the NAc

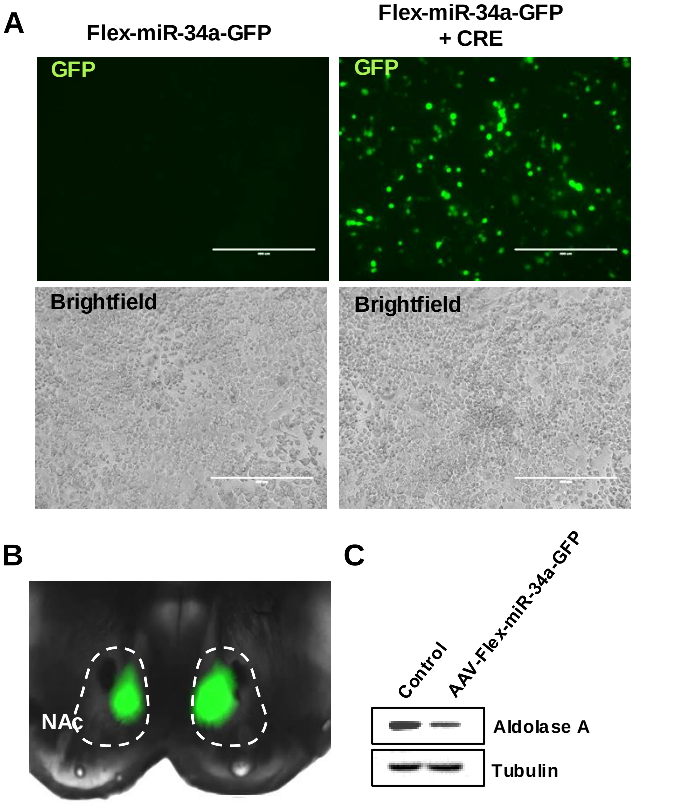

Supplementary Figure 12 Subcutaneous administration of NaCl does not affect alcohol consumption

Mice underwent 7 weeks of IA20%2BC (**Supplementary Table 1**) whereas control animals had access to water bottles only. On weeks 10 and 11, mice were s.c. injected with NaCl (1g/kg) or PBS in a counterbalanced manner 30 minutes before the beginning of a 24-hour drinking session. Alcohol and water consumption was measured at 4 hours (**A-C**) and 24 hours (**D-F**) timepoints. Data are presented as individual data points and mean ± SEM. *$p<0.05$, **$p<0.01$. ns: non-significant. $n = 9$ per group. Significance was determined using two-tailed paired t-tests. (**A**) $t(8)=0.4397$, $p=0.6718$; (**B**) $t(8)=4.204$, $p=0.003$; (**C**) $t(8)=2.613$, $p=0.031$; (**D**) $t(8)=0.5005$, $p=0.6302$; (**E**) $t(8)=0.1377$, $p=0.8938$; (**F**) $t(8)=0.3203$, $p=0.7569$.

Source data are provided as a Source Data file.

Supplementary Figure 12: Subcutaneous administration of NaCl does not affect alcohol consumption

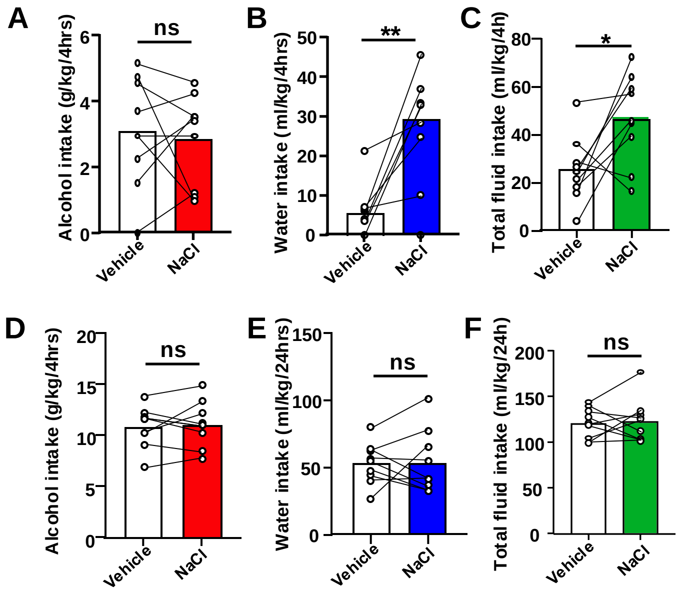

Supplementary Figure 13 Validation of the RiboTag technique

Polysomal mRNA levels in D1+ vs. D2+ neurons of Drd1 (**A**) and Drd2 (**B**) were determined by RT-qPCR. Data are presented as the average ratio of a transcript to GAPDH±SEM and expressed as % of water control. *$p<0.05$. D1+: $n = 5$; D2+: $n = 4$. Significance was determined using two-tailed Mann-Whitney t-test. (**A**) $U=0$ $p=0.0159$; (**B**) $U=0$, $p=0.0159$;

Source data are provided as a Source Data file.

Supplementary Figure 13: Validation of the RiboTag technique

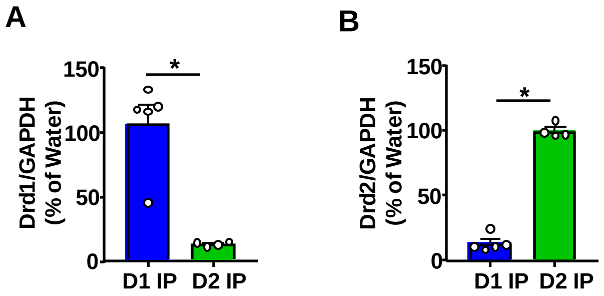

Supplementary Table 1 Alcohol drinking averages of mouse groups used in the study

Shown are average alcohol intake ±SEM of the last 3 24-hours sessions for each of the experimental groups.

Supplementary Table 1: Alcohol and sucrose drinking averages

Alcohol	Average Consumption (g/kg/24hr)	SEM (±g/kg/24hr)
**1B-D and 2A-C**	12.14	1.14
**1E-H, 2D-I, S1C-D, S2C-F and S4D-I,**	12.52	1.63
**3G-L, S1A, S14A**	17.89	1.47
**4B and S7**	13.25	0.97
**6B**	14.62	0.95
**6C**	11.30	0.87
**7B-G and S12**	10.88	2.62
**S2A-B and S4A-C**	13.60	0.96
**4C-D**	12.55	1.37
**S1B, S5, S14B**	16.41	1.17
**Sucrose**	**Average Consumption (ml/kg/24hr)**	**SEM (±ml/kg/24hr)**
**S13**	213.56	21.07

Supplementary Table 2 Statistical analyses of the data

Shown are figure number, number of animals or replicates, statistical test and statistical analysis.

Supplementary Table 2 Statistical analyses of the data

	Group Size	Statistical Test	Main effect	t- or f-statistic	p-value	Post-hoc	Post-hoc comparison	p-value
				F(DFn, DFd)				
Figure 1B	n=5 per group	Two-way ANOVA	Alcohol x Rapamycin	F(1, 16)=13.86	p=0.0019	Tukey's test	water and alcohol within the vehicle group	p=0.0008
			Alcohol	F(1, 16)=10.23	P=0.0056		vehicle and rapamycin within the alcohol group	p=0.0014
			Rapamycin	F(1, 16)=7.957	P=0.0123			
Figure 1C	n=5 per group	Two-way ANOVA	Alcohol x Rapamycin	F(1, 16)=6.541	p=0.0211	Tukey's test	water and alcohol within the vehicle group	p=0.0137
			Alcohol	F(1, 16)=5.84	P=0.0280		vehicle and rapamycin within the alcohol group	p=0.0165
			Rapamycin	F(1, 16)=5.224	P=0.0363			
Figure 1D	n=5 per group	Two-way ANOVA	Alcohol x Rapamycin	F (1, 16) = 2.198	P=0.1576			
			Alcohol	F(1, 16)=0.1552	p=0.6989			
			Rapamycin	F(1, 16)=0.0019	p=0.9656			
Figure 1G	n=8 per group	Unpaired t-test		t(14)=4.354	p=0.0007			
Figure 1H	n=8 per group	Unpaired t-test		t(14)=4.354	p=0.0007			
Figure 2A	n=5 per group	Two-way ANOVA	Alcohol x Rapamycin	F(1, 16)=18.01	p=0.0006	Tukey's test	water and alcohol within the vehicle group	p=0.0001
			Alcohol	F(1, 16)=16.38	P=0.0009		vehicle and rapamycin within the alcohol group	p=0.0001
			Rapamycin	F(1, 16)=15.56	P=0.0012			
Figure 2B	n=5 per group	Two-way ANOVA	Alcohol x Rapamycin	F(1, 16)=12.86	p=0.0025	Tukey's test	water and alcohol within the vehicle group	p=0.001
			Alcohol	F(1, 16)=10.51	P=0.0051		vehicle and rapamycin within the alcohol group	p=0.0002
			Rapamycin	F(1, 16)=19.13	P=0.0005			
Figure 2C	n=5 per group	Two-way ANOVA	Alcohol x Rapamycin	F(1, 16)=27.39	p<0.0001	Tukey's test	water and alcohol within the vehicle group	p<0.0001
			Alcohol	F(1, 16)=18.9	P=0.0005		vehicle and rapamycin within the alcohol group	p<0.0001
			Rapamycin	F(1, 16)=11	P=0.0044			
Figure 2G	n=8 per group	Unpaired t-test		t(14)=4.413	p=0.0006			
Figure 2H	n=8 per group	Unpaired t-test		t(14)=5.426	p<0.0001			
Figure 2I	n=8 per group	Unpaired t-test		t(14)=2.917	p=0.0113			
Figure 3D	n=3 per group	Unpaired t-test		t(4)=27.29	p=<0.0001			
Figure 3G	n=9-10 per group	Unpaired t-test		t(17)=4.042	p=0.0008			

Figure 3H	n=9-10 per group	Unpaired t-test		t(17)=2.575	p=0.0197			
Figure 3I	n=9-10 per group	Unpaired t-test		t(8)=2.322	p=0.0488			
Figure 3J	n=9-10 per group	Unpaired t-test		t(17)=2.299	p=0.0345			
Figure 3K	n=9-10 per group	Unpaired t-test		t(17)=3.425	p=0.0032			
Figure 3L	n=9-10 per group	Unpaired t-test		t(17)=4.26	p=0.0005			
Figure 4B	n=7 per group	Two-way ANOVA	Alcohol x miR	F (8, 110) = 3.994	P=0.0003	Sidak's test	miR15b-5p water vs alcohol	p=0.0147
			Alcohol	F(1, 110)=33.14	p<0.0001		miR 25-3p water vs alcohol	p=0.0402
			miR	F(8, 110)=2.428	P=0.0187		miR 34a-5p water vs alcohol	p=0.0007
							miR 92a-3p water vs alcohol	p<0.0001
							miR 127-3p water vs alcohol	p=0.9977
							miR 34a-3p water vs alcohol	p>0.9999
							miR 122-5p water vs alcohol	p=0.9707
							miR 15a-5p water vs alcohol	p=0.9998
							miR 19b-3p water vs alcohol	p=0.6109
Figure 4C	n=5-6 per group	Two-way ANOVA	Alcohol x Rapamycin	F(1, 17)=7.291	p=0.0152	Sidak's test	water and alcohol within the vehicle group	p=0.0189
			Alcohol	F(1, 17)=5.107	P=0.0372		vehicle and rapamycin within the alcohol group	p=0.0366
			Rapamycin	F(1, 17)=2.621	P=0.1238			
Figure 4D	n=5-6 per group	Two-way ANOVA	Alcohol x Rapamycin	F(1, 17)=0.01218	P=0.9134			
			Alcohol	F(1, 17)=2.866	P=0.1087			
			Rapamycin	F(1, 17)=0.002773	P=0.9586			
Figure 5C	n=4 of independent replicates	Two-way ANOVA	miR vs. Aldolase A 3'UTR	F(2, 18)=7.882	p=0.0035	Tukey's test	Aldolase A 3'UTR alone vs. Aldolase A 3'UTR + miR34a-5p	p=0.0001
			Aldolase A 3'UTR	F(1, 18)=3.144	P=0.0931		Aldolase 3'UTR + miR34a-5p vs. Aldolase A 3'UTR + miR control	p<0.0001
			miR	F(2, 18)=34.75	P<0.0001		Aldolase A 3'UTR mutant + miR34a-5p vs. Aldolase A 3'UTR + miR34a-5p	p=0.0053
							Aldolase A 3'UTR + miR34a-5p vs. Aldolase A 3'UTR mutant + miR control	p<0.0001

Figure 5F	n=4-5 per group	Mann-Whitney test		U=0	p=0.0159			
Figure 6B	n= 6-7 per group	Mann-Whitney tests		Lactate U=4	p=0.0140			
				Citrate U=6	p=0.035			
				a-Ketogluturate U=6	p=0.035			
				Malate U=3	p=0.0082			
Figure 6C	n=6 per group	One-way ANOVA		F(2, 15)=13.48	p=0.0004	Tukey’s test	water/vehicle vs alcohol/vehicle	p=0.0005
							alcohol/vehicle vs alcohol/rapamycin	p=0.004
Figure 7B	n=9-10 per group	Two-way ANOVA Mixed-effects	miR34a-5p overexpression x Time	F (5, 84) = 1.036	p=0.4017	Sidak’s test	Session 1	p=0.0037
			miR34a-5p overexpression	F (1, 17) = 16.46	p=0.0008		Session 2	p<0.0001
			Time	F (5, 84) = 3.698	p=0.0045		Session 3	p=0.0578
							Session 4	p=0.0089
							Session 5	p=0.0277
							Session 6	p=0.061
Figure 7C	n=9-10 per group	Two-way ANOVA RM	miR34a-5p overexpression x Time	F (5, 85) = 0.3717	p=0.8667			
			miR34a-5p overexpression	F (5, 85) = 0.3105	p=0.5846			
			Time	F (5, 85) = 1.623	p=0.1627			
Figure 7D	n=9-10	Two-way RM ANOVA	Virus x Time	F (19, 323) = 2.614	P=0.0003			
			Virus	F (1, 17) = 1.148	P=0.2990			
			Time	F (17, 323) = 10.58	P<0.0001			
Figure 8A	n=9 per group	Paired t-test		t(8)=7.562	p<0.0001			
Figure 8B	n=9 per group	Paired t-test		t(8)=5.917	p=0.0004			
Figure 8C	n=9 per group	Paired t-test		t(8)=4.742	p=0.0015			
Figure 8D	n=9 per group	Paired t-test		t(8)=2.004	p=0.08			
Figure 8E	n=9 per group	Paired t-test		t(8)=2.352	p=0.0509			
Figure 8F	n=9 per group	Paired t-test		t(8)=1.316	p=0.2297			
Figure 8G	n=9 per group	paired t-test		t(8)=0.1349	p=0.8960			
Figure 8H	n=9 per group	paired t-test		t(8)=0.1971	p=0.8482			
Figure 8I	n=9 per group	paired t-test		t(8)=0.1370	p=0.8944			
Figure 8J	n=9 per group	paired t-test		t(8)=0.2076	p=0.8402			
Figure 8K	n=9 per group	paired t-test		t(8)=1.827	p=0.1051			

Figure 8L	n=9 per group	paired t-test		t(8)=0.4631	p=0.6556			
Figure 8M	n=9	Two-way RM ANOVA	Lactate x Time	F (19, 160) = 1.250	P=0.2247			
			L-lactate	F (19, 160) = 0.9351	P=0.5409			
			Time	F (1, 160) = 39.64	P<0.0001			
Figure S1A	n=4 per group	Mann-Whitney test		U=5	p=0.1508			
Figure S1B	n=4 per group	Mann-Whitney test		U=5	p=0.2			
Figure S1D	n=6 per group	Mann-Whitney test		U=9	p=0.1797			
Figure S2A	n=7 per group	Mann-Whitney test		U=18	p=0.4557			
Figure S2B	n=7 per group	Mann-Whitney test		U=17	p=0.3829			
Figure S2E	n=5-6 per group	Mann-Whitney test		U=16	p=0.8182			
Figure S2F	n=5-6 per group	Mann-Whitney test		U=12	p=0.6623			
Figure S4A	n = 7 per group	Mann-Whitney test		U=19	p=0.535			
Figure S4B	n = 7 per group	Mann-Whitney test		U=22	p=0.8048			
Figure S4C	n = 7 per group	Mann-Whitney test		U=22	p=0.8048			
Figure S4G	n=6 per group	Mann-Whitney test		U=13	p=0.4848			
Figure S4H	n=6 per group	Mann-Whitney test		U=8	p=0.132			
Figure S4I	n=6 per group	Mann-Whitney test		U=14	p=0.5887			
Figure S5A	n=4 per group	Mann-Whitney test		U=6	p=0.6857			
Figure S5B	n=4 per group	Mann-Whitney test		U=6	p=0.0571			
Figure S5C	n=4 per group	Mann-Whitney test		U=6	p=0.6857			
Figure S5D	n=4 per group	Mann-Whitney test		U=6	p=0.0286			
Figure S5E	n=4 per group	Mann-Whitney test		U=6	p=0.0571			
Figure S5F	n=4 per group	Mann-Whitney test		U=6	p=0.6857			
Figure S6	n=12 per group	Mann-Whitney test		U=65	p=0.7125			
Figure S7	n = 7 per group	Two-way ANOVA	Alcohol x miR	F (3, 48) = 0.1223	P=0.9465	Sidak's test	miR15b-5p water vs alcohol	p=0.8127
			Alcohol	F (1, 48) = 4.160	P=0.0469		miR 25-3p water vs alcohol	p=0.9047
			miR	F (3, 48) = 0.2028	P=0.8939		miR 34a-5p water vs alcohol	p= 0.8868

							miR 92a-3p water vs alcohol	p=0.4289
Figure S8	n=3 per group	Mann-Whitney test		U=1	p=0.2			
Figure S9A	n=3 per group	Mann-Whitney test		U=1	p=0.2			
Figure S9B	n=3 per group	Mann-Whitney test		U=2	p=0.4			
Figure S9C	n=3 per group	Mann-Whitney test		U=2	p=0.4			
Figure S9D	n=3 per group	Mann-Whitney test		U=2	p=0.4			
Figure S10A	n=6-12 per group	Two-way ANOVA	Alcohol x Time	F(4, 64)=2.232	p=0.0753			
			Alcohol	F(1, 16)=2.397	p=0.1411			
			Time	F(4, 64)=158.3	p<0.0001			
Figure S10B	n=6-12 per group	Two-way ANOVA	Alcohol x Time	F(4, 64)=1.911	p=0.1193			
			Alcohol	F(1, 16)=3.159	p=0.0945			
			Time	F(4, 64)=148.8	p<0.0001			
Figure S12A	n=9 per group	paired t-test		t(8)=0.4397	p=0.6718			
Figure S12B	n=9 per group	paired t-test		t(8)=4.204	p=0.003			
Figure S12C	n=9 per group	paired t-test		t(8)=2.613	p=0.031			
Figure S12D	n=9 per group	paired t-test		t(8)=0.5005	p=0.6302			
Figure S12E	n=9 per group	paired t-test		t(8)=0.1377	p=0.8938			
Figure S12F	n=9 per group	paired t-test		t(8)=0.3203	p=0.7569			
Figure S13A	n=4-5 per group	Mann-Whitney test		U=0	p=0.0159			
Figure S13B	n=4-5 per group	Mann-Whitney test		U=0	p=0.0159			

Supplementary Table 3 Alcohol decreases the translation of transcripts in an mTORC1-dependent manner

After 7 weeks of IA20%2BC, the NAc of mice was dissected at the end of the 24 hours alcohol withdrawal session. Control animals had access to water only. RNAseq data [19] are sorted in ascending order of fold change of alcohol+vehicle divided by water+vehicle with negative values indicating decreased translation by alcohol. Positive fold change of alcohol+vehicle divided by alcohol+rapamycin indicates a reversal of translation attenuation by rapamycin. Significance was determined using two-tailed unpaired t-test. n = 3.

Supplementary Table 3: Alcohol via mTORC1 decreases the translation of transcripts in the NAc

Gene ID	Gene Accession Number	Gene Name	Folds Alcohol	p value Alcohol	Folds Rapamycin	p value Rapamycin
Vwa1	NM_147776	von Willebrand factor A domain containing 1	-0.602	0.016	0.614	0.04
Dennd3	NM_001081066	DENN domain-containing protein 3	-0.589	0.002	0.655	0.039
Igtp	NM_018738	interferon gamma induced GTPase	-0.571	0.006	0.511	0.02
Riok2	NM_025934	RIO kinase 2	-0.563	0.038	0.489	0.024
Aldoa	NM_001177307	aldolase A	-0.557	0.022	0.716	0.038
Ptgs2	NM_011198	prostaglandin endoperoxide synthase 2	-0.55	0.031	0.664	0.045
Bhlha9	NM_177182	basic helix loop helix family member a9	-0.545	0.017	0.702	0.015
Gm5128	NM_183320	predicted gene 5128	-0.53	0.025	0.618	0.053
Hs3st4	NM_001252072	heparan sulfate 3-O-sulfotransferase 4	-0.506	0.008	0.403	0
Abi3	NM_025659	ABI gene family member 3	-0.467	0.014	0.368	0.03
Atxn7l3	NM_001098836	ataxin 7-like 3	-0.467	0.038	0.357	0.006
Mta3	NM_054082	metastasis associated 3	-0.465	0.014	0.503	0.03
Tsc1	NM_022887	tuberous sclerosis 1	-0.453	0.032	0.424	0.033
Neil 1	NM_028347	nei endonuclease VIII-like 1	-0.422	0.032	0.462	0.02
Robo3	NM_001164767	roundabout guidance receptor 3	-0.417	0.022	0.609	0.024
Pde4dip	NM_001039376	phosphodiesterase 4D interacting protein	-0.394	0.047	0.341	0.011
Ppm1e	NM_177167	protein phosphatase 1E	-0.386	0.054	0.658	0.004
Wbscr27	NM_024479	Williams Beuren syndrome chromosome region 27	-0.376	0.018	0.361	0.011
Clgn	NM_009904	calmegin	-0.376	0.018	0.418	0.034
Zranb3	NM_027678	zinc finger RNA-binding domain containing 3	-0.362	0.02	0.423	0.032
Tyw5	NM_001037742	tRNA-yW synthesizing protein 5	-0.35	0.034	0.316	0.019
Flt1	NM_010228	FMS-like tyrosine kinase 1	-0.315	0.01	0.41	0.047
Fam163b	NM_175427	protein fam163b	-0.286	0.001	0.307	0.047
Ccdc93	NM_001025156	coiled-coil domain containing 93	-0.23	0.012	0.224	0.03
Epb41l4a	NM_013512	erythrocyte membrane protein band 4.1 like 4a	-0.213	0.04	0.246	0.047
Pdlim1	NM_016861	PDZ and Lim domain 1	-0.206	0.021	0.235	0.031
Rbfox2	NM_175387	RNA binding protein fox1 homolog	-0.182	0.018	0.199	0.041
Tsen34	NM_001164204	tRNA splicing endonuclease subunit 34	-0.181	0.013	0.268	0.015
Xirp2	NM_001083919	xin actin binding repeat containing 2	-0.169	0.004	0.459	0.034
Lcn2	NM_008491	lipocalin	-0.165	0.019	0.087	0.036
Inip	NM_001013577	INTS3 and NABP interacting protein	-0.152	0.012	0.169	0.005
Klhl3	NM_001195075	kelch-like protein 3	-0.062	0.023	0.084	0.041

Supplementary Table 4 List of primers used in the study

Shown are genes and miR names and corresponding primer information used for RT-qPCR.

Supplementary Table 4: List of primers used in the study

Gene or miR	Source	Sequence or reference
Trax	In house	F: GCTGGATGGTGTCAGACAGA; R: GAAAAGAGACAGCCTCCACG
GW182	In house	F: GCAGGGATTTAGTGCAAGAAG; R: GTGGAAGTGCCGTTATCAG
PPM1E	In house	F: GCCAGAGCCACATCAGATGA; R: AAGTCCTTCACCCAACTGCA
Aldolase A	In house	F: TAGCCGCGTTCGCTCCTTAG; R: CCTTCTTCTGCTCCGGGGTC
Rbfox2	In house	F: TCCGAGGAGACCATCTGAGG; R: AATCCGTCCTGGTAAACCACA
GAPDH	In house	F: CGACTTCAACAGCAACTCCCACTCTTCC; R: TGGGTGGTCCAGGGTTTCTTACTCCTT
miR15b-5p	Qiagen	YP00204243
miR25-3p	Qiagen	YP00204361
miR34a-5p	Qiagen	YP00204486
miR92a-3p	Qiagen	YP00205947
miR127-3p	Qiagen	YP00204048
miR34a-3p	Qiagen	YP02108859
U6 snRNA	Qiagen	YP00203907

Supplementary Table 5 List of antibodies used in the study

Shown are antibodies, references, and manufacturers.

Supplementary Table 5: List of antibodies used in the study

**Antibodies**			Dilution
Rabbit anti-Aldolase A	Cell Signaling	3188s	1:2000
Rabbit anti-PPM1E	Abnova	PAB21197	1:2000
Rabbit anti-Rbfox2	Bethyl	A300-864A	1:500
Rabbit anti-Trax	Abgent	AP13947a	1:1000
Rabbit anti-GW182	Sigma	SAB2102506-100UL	1:500
Mouse IgM anti-Tubulin	Santa Cruz Biotechnology	SC-8035	1:10,000
Rabbit anti-Aldolase C	Cell Signaling	81944s	1:1000
Mouse anti-GAPDH	Sigma	G8795	1:10,000
Rabbit anti-phospho S6 ribosomal protein	Cell Signaling	2211s	1:500
Guinea pig anti-NeuN	Millipore	ABN90	1:500
Donkey anti-rabbit horseradish peroxidase	Jackson ImmunoResearch	711-035-152	1:5000
Donkey anti-mouse horseradish peroxidase	Jackson ImmunoResearch	715-035-150	1:5000
Goat anti-mouse IgM horseradish peroxidase	Jackson ImmunoResearch	115-035-020	1:5000
Donkey anti Rabbit Alexa fluor 488	Thermo Fisher Scientific	A21206	1:1000
Goat anti Guinea Pig Alexa fluor 594	Thermo Fisher Scientific	A11076	1:1000
Anti-GFP	Memorial Sloan-Kettering Monoclonal Antibody Facility	clone 19C8 and clone 19F7	
