# Supplementary material for: Paradoxical mTORC1-Dependent microRNA-mediated Translation Repression in the Nucleus Accumbens of Male Mice Consuming Alcohol Attenuates Glycolysis
Source: Nat Commun. 2025 Jul 14;16:6116. doi: 10.1038/s41467-025-60337-9 (PMC12259992; doi:10.1038/s41467-025-60337-9)
Supplement: Supplementary file 2 — Reporting Summary [file 41467_2025_60337_MOESM2_ESM.pdf]

nature portfolio

Corresponding author(s): Dorit Ron

Last updated by author(s): Apr 2, 2025

Reporting Summary

Nature Portfolio wishes to improve the reproducibility of the work that we publish. This form provides structure for consistency and transparency in reporting. For further information on Nature Portfolio policies, see our Editorial Policies and the Editorial Policy Checklist.

Statistics

For all statistical analyses, confirm that the following items are present in the figure legend, table legend, main text, or Methods section.

n/a	Confirmed	
☐	☒	The exact sample size (*n*) for each experimental group/condition, given as a discrete number and unit of measurement
☐	☒	A statement on whether measurements were taken from distinct samples or whether the same sample was measured repeatedly
☐	☒	The statistical test(s) used AND whether they are one- or two-sided<br>*Only common tests should be described solely by name; describe more complex techniques in the Methods section.*
☒	☐	A description of all covariates tested
☐	☒	A description of any assumptions or corrections, such as tests of normality and adjustment for multiple comparisons
☐	☒	A full description of the statistical parameters including central tendency (e.g. means) or other basic estimates (e.g. regression coefficient) AND variation (e.g. standard deviation) or associated estimates of uncertainty (e.g. confidence intervals)
☐	☒	For null hypothesis testing, the test statistic (e.g. *F*, *t*, *r*) with confidence intervals, effect sizes, degrees of freedom and *P* value noted<br>*Give P values as exact values whenever suitable.*
☒	☐	For Bayesian analysis, information on the choice of priors and Markov chain Monte Carlo settings
☒	☐	For hierarchical and complex designs, identification of the appropriate level for tests and full reporting of outcomes
☐	☒	Estimates of effect sizes (e.g. Cohen's *d*, Pearson's *r*), indicating how they were calculated

Our web collection on statistics for biologists contains articles on many of the points above.

Software and code

Policy information about availability of computer code

Data collection	PRISM software (Graphpad, version 8); Excel (Microsoft); ethovision XT 17, ProteoWizard, Maven (v 8.1.27.11)
Data analysis	PRISM software (Graphpad, version 8)

For manuscripts utilizing custom algorithms or software that are central to the research but not yet described in published literature, software must be made available to editors and reviewers. We strongly encourage code deposition in a community repository (e.g. GitHub). See the Nature Portfolio guidelines for submitting code & software for further information.

Data

Policy information about availability of data

All manuscripts must include a data availability statement. This statement should provide the following information, where applicable:

- Accession codes, unique identifiers, or web links for publicly available datasets
- A description of any restrictions on data availability
- For clinical datasets or third party data, please ensure that the statement adheres to our policy

The authors declare that all relevant data supporting the findings of this study are included in this published article, supplementary information files and source data file. The metabolomic data generated in this study have been deposited in the Zenodo database under accession code https://doi.org/ 10.5281/zenodo.15178256. Source data are provided in this paper.

Research involving human participants, their data, or biological material

Policy information about studies with human participants or human data. See also policy information about sex, gender (identity/presentation), and sexual orientation and race, ethnicity and racism.

Reporting on sex and gender	*Use the terms sex (biological attribute) and gender (shaped by social and cultural circumstances) carefully in order to avoid confusing both terms. Indicate if findings apply to only one sex or gender; describe whether sex and gender were considered in study design; whether sex and/or gender was determined based on self-reporting or assigned and methods used. Provide in the source data disaggregated sex and gender data, where this information has been collected, and if consent has been obtained for sharing of individual-level data; provide overall numbers in this Reporting Summary. Please state if this information has not been collected. Report sex- and gender-based analyses where performed, justify reasons for lack of sex- and gender-based analysis.*
Reporting on race, ethnicity, or other socially relevant groupings	*Please specify the socially constructed or socially relevant categorization variable(s) used in your manuscript and explain why they were used. Please note that such variables should not be used as proxies for other socially constructed/relevant variables (for example, race or ethnicity should not be used as a proxy for socioeconomic status). Provide clear definitions of the relevant terms used, how they were provided (by the participants/respondents, the researchers, or third parties), and the method(s) used to classify people into the different categories (e.g. self-report, census or administrative data, social media data, etc.) Please provide details about how you controlled for confounding variables in your analyses.*
Population characteristics	*Describe the covariate-relevant population characteristics of the human research participants (e.g. age, genotypic information, past and current diagnosis and treatment categories). If you filled out the behavioural & social sciences study design questions and have nothing to add here, write "See above."*
Recruitment	*Describe how participants were recruited. Outline any potential self-selection bias or other biases that may be present and how these are likely to impact results.*
Ethics oversight	*Identify the organization(s) that approved the study protocol.*

Note that full information on the approval of the study protocol must also be provided in the manuscript.

Field-specific reporting

Please select the one below that is the best fit for your research. If you are not sure, read the appropriate sections before making your selection.

☒ Life sciences ☐ Behavioural & social sciences ☐ Ecological, evolutionary & environmental sciences

For a reference copy of the document with all sections, see nature.com/documents/nr-reporting-summary-flat.pdf

Life sciences study design

All studies must disclose on these points even when the disclosure is negative.

Sample size	No statistical methods were explicitly used for predetermination of sample size. Sample sizes were similar those used in previous publications (Laguesse et al. Neuron, 2017, Morisot et al. Addiction Biology , 2018, Laguesse et al. Neuropsychopharmacology, 2018).
Data exclusions	No mouse was excluded. Two data points in drinking experiments are missing due to bottle spillage.
Replication	All molecular and biochemical experiments were replicated at least in two independent mouse cohorts. Behavioral experiments were performed in one cohort of mice.
Randomization	Mice are assigned to each experimental groups based on random selection.
Blinding	For western blot and rt-qPCR, the same investigators both designed and performed experiments , therefore no blinding concerning sample identity for data collection but analysis was blinded. Imaging and counting were blinded. Metabolomic anlalysis was blinded. Behavioral experiments were performed blinded to the conditions (collection and analysis).

Reporting for specific materials, systems and methods

We require information from authors about some types of materials, experimental systems and methods used in many studies. Here, indicate whether each material, system or method listed is relevant to your study. If you are not sure if a list item applies to your research, read the appropriate section before selecting a response.

Materials & experimental systems

n/a	Involved in the study
☐	☒ Antibodies
☐	☒ Eukaryotic cell lines
☒	☐ Palaeontology and archaeology
☐	☒ Animals and other organisms
☒	☐ Clinical data
☒	☐ Dual use research of concern
☒	☐ Plants

Methods

n/a	Involved in the study
☒	☐ ChIP-seq
☒	☐ Flow cytometry
☒	☐ MRI-based neuroimaging

Antibodies

Antibodies used

Catalogue number, lot number and dilution used are listed in Supplementary Table 5.
Antibodies:
Rabbit anti-Aldolase A Cell Signaling 3188s 1:2000
Rabbit anti-PPM1E Abnova PAB21197 1:2000
Rabbit anti-Rbfox2 Bethyl A300-864A 1:500
Rabbit anti-Trax Abgent AP13947a 1:1000
Rabbit anti-GW182 Sigma SAB2102506-100UL 1:500
Mouse IgM anti-Tubulin Santa Cruz Biotechnology SC-8035 1:10,000
Rabbit anti-Aldolase C 1:1000
Mouse anti-GAPDH Sigma G8795 1:10,000
Rabbit anti-phospho S6 ribosomal protein Cell Signaling 2211s 1:500
Guinea pig anti-NeuN Millipore ABN90 1:500
Donkey anti-rabbit horseradish peroxidase Jackson ImmunoResearch 711-035-152 1:5000
Donkey anti-mouse horseradish peroxidase Jackson ImmunoResearch 715-035-150 1:5000
Goat anti-mouse IgM horseradish peroxidase Jackson ImmunoResearch 115-035-020 1:5000
Donkey anti Rabbit Alexa fluor 488 Thermo Fisher Scientific A21206 1:1000
Goat anti Guinea Pig Alexa fluor 594 Thermo Fisher Scientific A11076 1:1000
Anti-GFP Memorial Sloan-Kettering Monoclonal Antibody Facility clone 19C8 and clone 19F7

Validation

All antibodies were purchased from qualified vendors that provide validation on the website of the manufacturer.
We validated each of the antibodies in house and made sure that the band of the protein is at the right molecular weight.

Eukaryotic cell lines

Policy information about cell lines and Sex and Gender in Research

Cell line source(s)

All cell lines were purchased from qualified vendors. HEK293 were purchased from ATCC and HEK293 lentiX were purchased from Clontech.

Authentication

None of the cell lines used were authenticated.

Mycoplasma contamination

All cell lines tested negative for mycoplasma (Mycostrip, InvivoGen).

Commonly misidentified lines
(See ICLAC register)

Name any commonly misidentified cell lines used in the study and provide a rationale for their use.

Animals and other research organisms

Policy information about studies involving animals; ARRIVE guidelines recommended for reporting animal research, and Sex and Gender in Research

Laboratory animals

Male C57BL/6J mice (Jackson Laboratory, Bar Harbor, ME), Drd1a-Cre (D1-Cre) and AdoraA2-Cre (A2A-Cre) mice both of which are on C57BL/6 background, (Mutant Mice Resource and Research Centers (MMRRC) UC Davis) and Ribotag mice (ROSA26CAGGFP-L10a), (The Jackson Laboratory (B6;129S4-Gt (ROSA)26Sortm9(EGFP/Rpl10a)Amc/J)) were 6-7 weeks old at the beginning of the experiments. Mice were individually housed in separate temperature- and humidity- controlled rooms (temperature and humidity were kept constant at 22 ± 2°C, and relative humidity was maintained at 50 ± 5%) under a 12-hour light/dark cycle (lights on at 07:00 AM) or a reversed 12 hour light/dark cycle (lights on at 10:00 PM) with food and water available ad libitum.

Wild animals

The study did not include wild animals.

Reporting on sex

Our reasoning for examining this pathway in male mice stemmed from our findings that mTORC1 is not activated in adult female mice. Furthermore, inhibition of mTORC1 by systemic administration (1) or intra NAc infusion (2), of the selective mTORC1 inhibitor, rapamycin, does not alter alcohol intake in females. These data strongly suggest that the mTORC1-dependent pathway in the NAc neurons does not produce neuronal adaptations detected in male mice. To confirm this conclusion, we conducted an additional experiment in which we measured the level of Aldolase A protein levels in alcohol drinking vs. water drinking female mice. In contrast

to males, Aldolase A levels were unchanged by alcohol drinking in females (Rebuttal Figure 1). These data suggest that this signaling cascade is specifically relevant to males. However, it is plausible that mTORC1 is activated in females by alcohol in other regions and/or cell types. We are currently conducting a survey of alcohol-mediated mTORC1 activation patterns in female mice.

1 Ehinger, Y., Phamluong, K. & Ron, D. Sex differences in the interaction between alcohol and mTORC1. bioRxiv, 2023.2010.2004.560781, doi:10.1101/2023.10.04.560781 (2023)
2 Cozzoli, D. K. et al. Functional regulation of PI3K-associated signaling in the accumbens by binge alcohol drinking in male but not female mice. Neuropharmacology 105, 164-174, doi:10.1016/j.neuropharm.2016.01.010 (2016).

Field-collected samples

The study did not include samples collected from the field.

Ethics oversight

All animal procedures were approved by the University of California San Francisco (UCSF) Institutional Animal Care and Use Committee and conducted in agreement with the Association for Assessment and Accreditation of Laboratory Animal Care (AAALAC, UCSF).

Note that full information on the approval of the study protocol must also be provided in the manuscript.

Plants

Seed stocks

Report on the source of all seed stocks or other plant material used. If applicable, state the seed stock centre and catalogue number. If plant specimens were collected from the field, describe the collection location, date and sampling procedures.

Novel plant genotypes

Describe the methods by which all novel plant genotypes were produced. This includes those generated by transgenic approaches, gene editing, chemical/radiation-based mutagenesis and hybridization. For transgenic lines, describe the transformation method, the number of independent lines analyzed and the generation upon which experiments were performed. For gene-edited lines, describe the editor used, the endogenous sequence targeted for editing, the targeting guide RNA sequence (if applicable) and how the editor was applied.

Authentication

Describe any authentication procedures for each seed stock used or novel genotype generated. Describe any experiments used to assess the effect of a mutation and, where applicable, how potential secondary effects (e.g. second site T-DNA insertions, mosiacism, off-target gene editing) were examined.
